# Compact multi-foci metalens spectrometer

**DOI:** 10.1038/s41377-023-01148-9

**Published:** 2023-05-04

**Authors:** Ruoxing Wang, Muhammad Afnan Ansari, Hammad Ahmed, Yan Li, Wenfeng Cai, Yanjun Liu, Songtao Li, Jianlong Liu, Li Li, Xianzhong Chen

**Affiliations:** 1 Department of Mathematics and Physics, North China Electric Power University, 071003 Baoding, China; 2 Institute of Photonics and Quantum Sciences, School of Engineering and Physical Sciences, Heriot-Watt University, Edinburgh, EH14 4AS UK; 3 School of Materials, Zhengzhou University of Aeronautics, 450015 Zhengzhou, China; 4 Department of Electrical and Electronic Engineering, Southern University of Science and Technology, 518055 Shenzhen, China; 5 College of Physics and Optoelectronic Engineering, Harbin Engineering University, 150001 Harbin, China; 6 School of Physics, Harbin Institute of Technology, 150001 Harbin, China

**Keywords:** Metamaterials, Nanophotonics and plasmonics

## Abstract

A lightweight and portable spectrometer is desirable for miniaturization and integration. The unprecedented capability of optical metasurfaces has shown much promise to perform such a task. We propose and experimentally demonstrate a compact high-resolution spectrometer with a multi-foci metalens. The novel metalens is designed based on wavelength and phase multiplexing, which can accurately map the wavelength information into its focal points located on the same plane. The measured wavelengths in the light spectra agree with simulation results upon the illumination of various incident light spectra. The uniqueness of this technique lies in the novel metalens that can simultaneously realize wavelength splitting and light focusing. The compactness and ultrathin nature of the metalens spectrometer render this technology have potential applications in on-chip integrated photonics where spectral analysis and information processing can be performed in a compact platform.

## Introduction

Variety of important optical phenomena (e.g., fluorescence or Raman scattering) and light matter interactions can be understood and explored using spectrometers. Therefore, spectrometers have been extensively used in many fields ranging from fundamental scientific research^1,2^ to disease diagnostics in the health sector^3,4^, food and drug safety^5^, and environmental monitoring^6^. Dispersive optical elements (e.g., a diffraction grating or a prism) are the key components of a spectrometer to achieve the desired dispersion along with focusing and detection. Dispersion originates from the variation of the material’s refractive index with the wavelength of the incident light beam. Like many spectrometric instruments, the accurate control of the dispersion is required in various fundamental research and practical applications such as state-of-the-art microscopes, metrology instruments, cameras, and pulse-spreading in optical fibers^7,8^.

Two types of dispersion control have been reported in the literature to either eliminate or enhance the dispersion in an optical system^7,9,10^. For example, the dispersion may cause polychromatic light beam to stray during the transmission, resulting in signal crosstalk and distortion in the optical communication systems. Moreover, it can introduce chromatic aberration in an imaging system and reduce the imaging quality, therefore, dispersion is minimized in such systems^10^. On the other hand, dispersion is enhanced to improve the spectral resolution of the spectral analyzers^11–13^. The desired dispersion control is challenging and indispensable in the modern optical designs and manufacturing processes. Conventional techniques to overcome this challenge require the combination of lenses and precisely machined components that are made of different materials^13,14^. The introduction of lenses with different materials can provide more degrees of freedom for the dispersion control of the entire system. However, tailoring dispersion with traditional methods (e.g., the combination of lenses and free-space optical components) is extremely challenging due to the accurate alignment. In addition, there exists a significant overhead in terms of bulkiness and complexity of the dispersion control system due to the large volume of free-space propagation. This poses a great challenge to meet the needs of the existing on-chip photonic integration for truly compact and handheld spectrometers.

Recently, there is a considerable interest in the high-resolution and compact spectrometers, which can be integrated with consumer electronics for applications such as quality control and material characterization^15,16^. To reduce the volume, a great effort has been made to develop compact spectrometers based on micro-optical components. These are conceptually like the traditional spectrometers, however, they possess lower resolution and limited micro-optical control with small optical path lengths^17^. High resolution can be achieved by introducing planar gratings (for the aberration correction) and an external spherical mirror, which make the spectrometer very bulky^18^.

In contrast, the ultrathin metasurface-based design has opened new frontiers for the miniaturization of optical devices in many fields such as metalenses^19–21^, holographic imaging^22–27^, and vortex beam generation^28–32^. Dispersive metasurfaces are capable to effectively achieve the dispersion control and management by tailoring the structural geometric parameters and arrangement of subwavelength nanostructures. As a result, the dispersion manipulation based on metasurfaces has been demonstrated to achieve broadband achromatic devices by minimizing dispersion^33–35^ and to enhance the dispersion for applications such as spectral analysis^6,36^. A single metasurface with the alternative phase matching technique can enable the ultra-dispersive diffraction^37^. Moreover, a metalens designed for a single wavelength can provide the required dispersion to distinguish spatial positions of the focal points which correspond to different incident wavelengths. A metalens with off-axis focal points can achieve the enhanced intrinsic dispersion without the shadowing effect, therefore, it can effectively diffract the light beam to larger angles compared to the conventional gratings^38,39^. However, the focal spots at different wavelengths have different focal sizes. A metalens designed for a single wavelength can only provide intrinsic dispersion at other wavelengths, but cannot control dispersion. Later, a sub-regional design based metalens was demonstrated to filter wavelengths and achieve an independent control of dispersion at three different working wavelengths^40^. However, the sub-regional design of the phase profile suffers from the limited number of pixels for each wavelength, which greatly decrease the quality of beam convergence. Moreover, it limits the possibility of a metalens to manipulate dispersion for more wavelengths. Furthermore, a new method is presented to design the optical group lengths obtained by different wavelengths of the light beam to achieve the dispersion manipulation^41^. Similarly, the concept of folded metasurface is introduced to demonstrate a compact spectrometer by using three or more reflective metasurfaces^36,42^. However, these methods are very complex as they require three or more metasurface structures to provide the required design freedom.

Unlike previous demonstrations, a metalens with multiple focal points was realized based on a single-phase profile design^43^. Theoretically, the number of focal points generated by a metalens designed in this way can be increased indefinitely. In our recent work, a metalens with a single-phase profile was demonstrated to generate 2000 focal points with an engineered polarization profile to create a three-dimensional (3D) polarization structure^19^. Subsequently, we utilized the intrinsic dispersion of a multi-foci metalens to realize color-selective 3D polarization structures^44^. Inspired by the intrinsic dispersion and multi-foci property of a metalens, the wavelength information can be accurately mapped to the intensity distribution of the focal points on the same plane. In this work, we experimentally tailor the dispersion using a single metasurface based on the multi-foci metalens model to include the wavelength information. The proposed metasurface spectrometer can split and focus light beams of different wavelengths at the predesigned positions on a focal plane with a high-resolution dispersion control under the illumination of both monochromatic and polychromatic light beams. The ultracompact spectrometer has achieved nanometer spectral resolution over a broadband visible domain at a working distance of 300 μm. Due to the novel design of multiwavelength multi-foci metalens, all focal points have nearly the same spot size with a maximum intensity on the designed focal plane, which has not been realized in the previously demonstrated metalens designs with off-axis focal points^38,39^. To the best of our knowledge, this work is the first experimental demonstration of multi-foci dispersion engineering using a single metasurface, offering a new approach for the implementation of an ultra-thin compact spectrometer. Furthermore, due to the easy fabrication of such ultra-thin devices with the possibility of integration with electronics and sensors, the proposed metadevice opens new frontiers in compact spectrometry applications (e.g., material characterization, disease diagnostics, and quality control) where stringent weight and volume constraint exist.

## Results

**Fig. 1:**
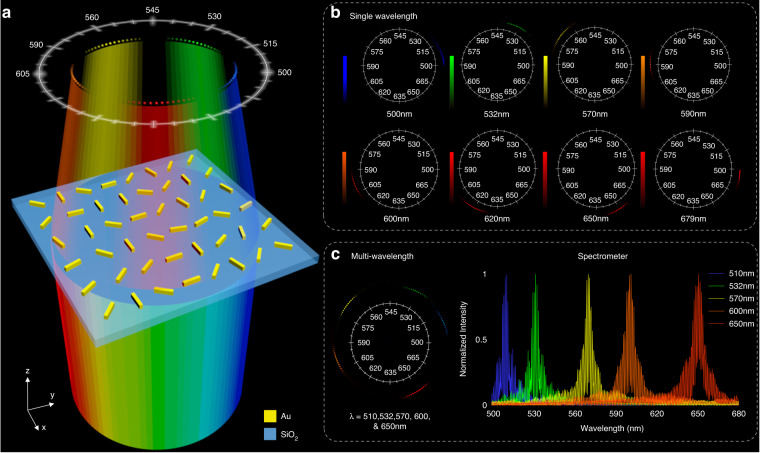
Schematic of the metasurface spectrometer based on a multi-foci metalens. **a** A schematic diagram of the proposed metasurface spectrometer based on wavelength dependent multi-foci metalens model. **b** Field distributions on the focal plane under the illumination of monochromatic incident light beams with different wavelengths. Light beams with different wavelengths converge at different positions on the focal plane. There exists one-to-one relationship between the azimuth angle of maximum intensity and the incident wavelength. **c** Field distribution on the focal plane under the illumination of polychromatic incident light beam (left panel). Five different wavelengths (510 nm, 532 nm, 570 nm, 600 nm, and 650 nm) of the incident light beams are taken as an example. By analyzing the normalized intensities on the ring, five central wavelengths of the polychromatic incident light beam are obtained (right panel)

Figure 1a presents the schematic of our proposed metasurface spectrometer that can split and focus the light beams with different wavelengths at different focal points on the same focal plane. The wavelengths of the incident polychromatic light beam are accurately mapped to different positions on the focal plane, achieving the functionality of a spectrometer. Our metasurface spectrometer consists of gold (Au) nanorods with spatially variant orientations sitting on a glass (SiO_2_) substrate. Upon the illumination of left circularly polarized (LCP) light, the right circularly polarized (RCP) light (converted part) is converged at the desired positions on a multi-foci ring. The ring is designed to incorporate multiple focal points, and each focal point corresponds to a different incident wavelength. As a result, the output light beam converges to a corresponding azimuth angle of the multi-foci ring in the case of a monochromatic incident light beam, as shown in Fig. 1b. The wavelength of the incident light beam is changed from 500 nm to 679 nm. There is more than one focal point on the focal plane for each monochromatic incident light beam. The calibration is carried out by analyzing the intensity profile on the focal plane with a predesigned ring chart, which is based on the focal points with the maximum intensity. The inclined angle *θ*_*h*_ that corresponds to the maximum intensity can be calculated using the expression *θ*_*h*_ = 2 × (*λ* − 500), where *λ* denotes the working wavelength of the incident light. The presence of low-intensity foci is due to the information of adjacent wavelengths in the phase profile of the metasurface. The left panel of Fig. 1c shows the case of a polychromatic incident light beam. Here, the proposed metasurface spectrometer is simultaneously illuminated with five different incident wavelengths, i.e., 510 nm, 532 nm, 570 nm, 600 nm, and 650 nm. As a result, five series of focal spots appear at the desired regions on the multi-foci ring. By analyzing the normalized intensity distribution on the ring, the desired central wavelengths of a polychromatic light beam can be obtained with high resolution, as shown in the right panel of Fig. 1c.

The design process of the proposed single-layer metasurface spectrometer includes a unique design technique of multi-foci metalens with wavelength information. To understand the underlying physical mechanism, we initially consider the phase profile of a metalens that can generate a single focal point at the desired position governed by Fermat’s principle^20^:

1 $$\varphi \left( {x,y} \right) = - \frac{{2\pi }}{{\lambda _0}}\left( {\sqrt {f^2 + \left( {x - x_0} \right)^2 + \left( {y - y_0} \right)^2} - f_D} \right)$$

where *λ*_0_ is the working wavelength, *f* is the position of the focal plane, $\left( {x_0,y_0} \right)$ are the coordinates of the focal point, and $f_D = \sqrt {f^2 + x_0^2 + y_0^2}$ is the distance from the focal point to the center of the metalens. Individual phase profiles of different metalenses with a single focal point can be integrated into one phase profile. The combined phase profile of the multi-foci metalens that can generate *N* focal points on the same focal plane can be expressed as^19^:

2 $$\Phi \left( {x,y} \right) = \arg \left\{ {\mathop {\sum }\limits_{j = 1}^N e^{i\varphi _j\left( {x,y} \right)}} \right\}$$

where $\varphi _j\left( {x,y} \right) = - \frac{{2\pi }}{{\lambda _0}}\left( {\sqrt {f^2 + \left( {x - x_j} \right)^2 + \left( {y - y_j} \right)^2} - f_{Dj}} \right)$ is the phase profile that generates the focal point with coordinates $\left( {x_j,y_j} \right)$, and $f_{Dj} = \sqrt {f^2 + x_j^2 + y_j^2}$.

Unlike previous designs, here, the wavelength of each focal point is considered a design variable to achieve the functionality of a spectrometer. A single-layer geometric metasurface is employed to realize the desired functionality. The metasurface can produce a Pancharatnam–Berry (PB) phase, which appears when the polarization state of light changes. The desired phase can be obtained by controlling the orientation angle *θ* of each nanorod. As a result, the local abrupt phase change is ±2*θ*, with the signs “+” and “−” for incident LCP and RCP light beams, respectively^19,20^. For the metasurface spectrometer, the wavelength information of each focal point is incorporated in the orientation angle profile. It will generate discrete multi-wavelength focal points, and the modified orientation angle profile can be written as:

3 $$\theta \left( {x,y} \right) = \frac{1}{2}\arg \left\{ {\mathop {\sum }\limits_{j = 1}^N e^{ - \frac{{2\pi i}}{{\lambda _j}}\left( {\sqrt {f^2 + \left( {x - x_j} \right)^2 + \left( {y - y_j} \right)^2} - f_{Dj}} \right)}} \right\}$$

where $\left( {x_j,y_j} \right)$ are the coordinates of the *j*th focal point on the focal plane with the working wavelength *λ*_*j*_.

**Fig. 2:**
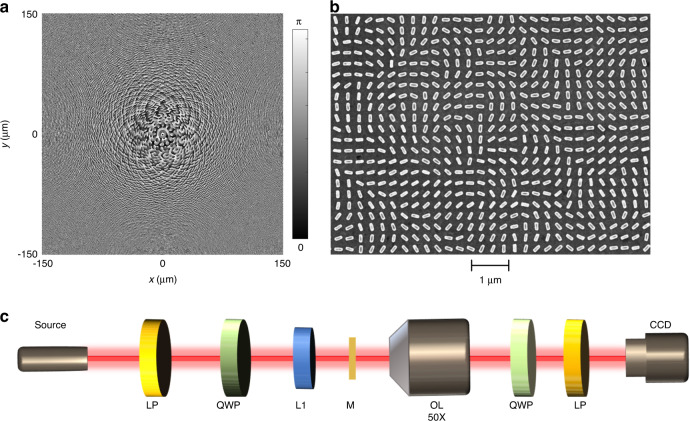
Design, fabrication and characterization of the metasurface spectrometer. **a** Nanorod orientation profile of the metasurface spectrometer with 12 focal points. **b** SEM image of the metasurface sample. **c** Schematic diagram of the experimental setup used to observe the field distributions on the focal plane

Off-axis focal points under the illumination of a light beam with different incident wavelengths are provided in Supplementary Section 1. Benefiting from the novel design of multiwavelength and multi-foci metalens governed by Eq. 3, it is noted that all focal points are approximately equal in size on the observation plane, with maximum intensities on the focal plane (Fig. S1). This functionality has not been realized in the previously demonstrated metalens designs with off-axis focal points^38,39^. Initially, 12 focal points (*N* = 12) are generated on a focal plane to map the corresponding working wavelengths for the proof of concept. The distance *f* between the focal plane and metalens is 300 μm. All focal points are placed on a ring with a radius of $r_0$ = 30 μm, where the coordinates of each focal point are $x_j = r_0\cos \frac{{j - 1}}{N}{{\Delta }}\alpha$ and $y_j = r_0\sin \frac{{j - 1}}{N}{{\Delta }}\alpha$, *j* is an integer with values from 1 to 12, and ${{\Delta }}\alpha = \frac{\pi }{6}$. The working wavelength of the *j*th focal point is expressed as:

4 $$\lambda _j = \lambda _0 + \left( {j - 1} \right){{\Delta }}\lambda$$

where $\lambda _0$ = 480 nm and ${{\Delta }}\lambda$ = 20 nm. With these parameters, the desired orientation angle profile can be obtained using Eq. 3, as illustrated in Fig. 2a. The metasurface spectrometer is experimentally realized based on a plasmonic metasurface, which consists of Au nanorods with different orientation angles *θ*(*x*, *y*) on a SiO_2_ substrate. Length (*l*), width (*w*), and height (*t*) of nanorods are 200 nm, 80 nm, and 40 nm, respectively. The periodicity (*p*) of each unit cell is 300 nm along both *x* and *y* directions as shown in Fig. S3a. Detailed information about the unit cell design is provided in Supplementary Section 2. The sample is fabricated using electron beam lithography for the desired orientation angle profile of nanorods. The total number of pixels in the sample is 1000 × 1000. The scanning electron microscopy (SEM) image of the fabricated metasurface spectrometer with 12 focal points is shown in Fig. 2b. The detailed fabrication process is available in the “Methods” section. Figure 2c shows the schematic diagram of the experimental setup. A tunable supercontinuum laser (NKT Photonics SuperK EXTREME) is used as a light source to characterize the metasurface spectrometer. The supercontinuum laser source can generate multiple wavelengths at a time. The phase profile of the metasurface spectrometer is designed for an incident LCP light beam. Therefore, to generate an incident LCP light beam, a linear polarizer (LP) and a quarter-wave plate (QWP) are used to control the incident polarization state. Later, the LCP light beam is focused on the sample through a lens (L1) to increase the power density of incident beam. The focal length of L1 is 100 mm, which can form a small cone angle of 0.57° in the incident beam. However, the influence on the field distribution is negligible due to the paraxial approximation. The effect of cone angle on the focusing performance at 660 nm is discussed in Supplementary Section 3. After the metasurface sample (M), an objective lens (OL) with a magnification of 50× is used to observe the field distribution of the light beam. Another pair of QWP and LP is used to eliminate the non-converted part. The intensity distribution on the focal plane is collected with a color CCD camera. The size of each focal point is constant under the same conditions with our current experimental setup. The size of focal point observed in the CCD depends on the magnification of the objective.

**Fig. 3:**
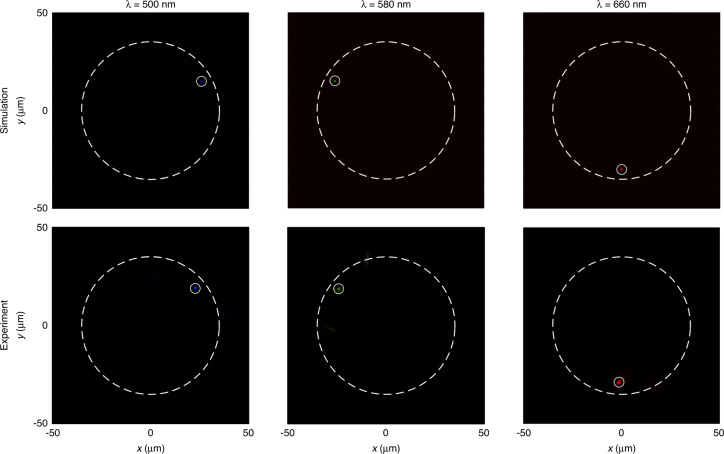
Metasurface spectrometer with 12 focal points under monochromatic wavelengths. The metasurface can accurately converge the light beam with different wavelengths to the desired positions on the multi-foci ring. Simulation (top) and experimental results (bottom) at the wavelengths of 500 nm (left), 580 nm (middle), and 660 nm (right)

Figure 3 shows the optical output of the metasurface spectrometer with a monochromatic incident light beam. The sample with 12 focal points is characterized with different wavelengths. The focal points appear at the desired positions with azimuth angles *π*/6, 5*π*/6, and 3*π*/2, which correspond to simulated 500 nm, 580 nm, and 660 nm wavelengths, respectively, as shown in the top row of Fig. 3. In the subplots of Fig. 3, each focal point is marked with a solid white circle. A dashed white circle of radius 35 μm is used as a reference for the boundary. The simulation results of the field distribution are calculated on the focal plane using the Fresnel–Kirchhoff diffraction integral. The experimental results are shown in the bottom row of Fig. 3, which agree with the predicted results. Results demonstrate the dispersion control capability of the designed device, which can accurately focus the light beams with different wavelengths to the desired positions. Additionally, a video is provided in Supplementary Section 4. The dynamic change of the focal points is observed as the incident wavelength is changed from 500 nm to 700 nm, which clearly shows that the proposed metasurface spectrometer can detect the wavelength change in the real time.

**Fig. 4:**
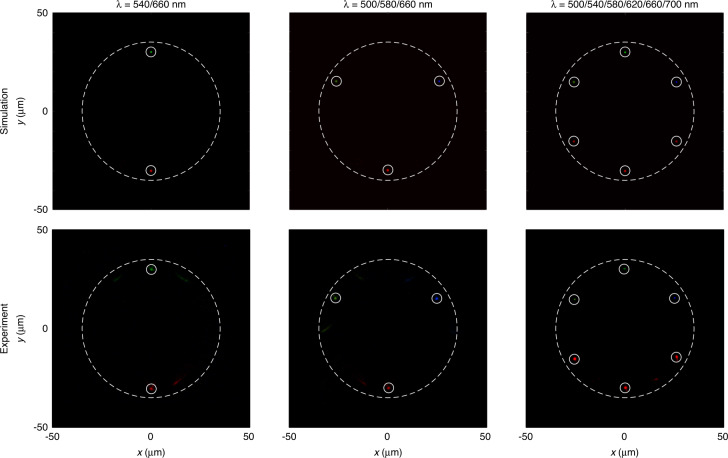
Metasurface spectrometer with 12 focal points under the polychromatic light beams. Simulation and experimental results under the different combination of wavelengths. Left: 540 nm and 660 nm, middle: 500 nm, 580 nm, and 660 nm, and right: 500 nm, 540 nm, 580 nm, 620 nm, 660 nm, and 700 nm

The metasurface spectrometer is characterized by a polychromatic incident light beam, as shown in Fig. 4. The results are presented for two, three, and six working wavelengths. When the light beam with different working wavelengths is used, the corresponding focal points appear accurately at the designed positions. The experimental results agree with the simulation results in the case of single and multiple wavelengths. It demonstrates the ability of our proposed metasurface spectrometer to detect the wavelengths accurately. Here, it is important to note that the resolution of the current design with 12 focal points is low. Moreover, the wavelength detection capability is not present for incident wavelengths away from the pre-designed wavelengths. The effect is demonstrated with more simulation and experimental results in Supplementary Section 5. When the incident wavelength is close to the middle of two adjacent designed wavelengths, the intensity distribution appears at the positions of two corresponding designed wavelengths. This effect is shown in Fig. S5a, b for 532 nm and 633 nm, respectively. When the incident wavelength is very close to a designed wavelength, the change in the distribution is negligible, as shown in Fig. S5c, f. This is due to the large intrinsic dispersion of the incident wavelength at the other designed focal positions.

A metasurface spectrometer with more working wavelengths is designed to solve the issue of low resolution. The new design consists of 180 focal points (*N* = 180), which correspond to 180 different wavelengths. The working wavelengths range from 500 nm to 679 nm with a wavelength spacing of 1 nm. The working wavelength of the *j*th focal point can be obtained using Eq. 4, where $\lambda _0$ = 500 nm, ${{\Delta }}\lambda$ = 1 nm, and *j* ranges from 1 to 180. The radius of the multi-foci ring $r_0$ is increased to 40 μm in order to achieve a higher dispersion difference between the focal positions of adjacent wavelengths. The increase in the radius can improve the wavelength detection accuracy. The effect of different radii on the dispersion difference is provided in Supplementary Section 6. The coordinates of each focal point on the ring can be expressed as $x_j = r_0\cos \frac{{j - 1}}{N}{{\Delta }}\alpha$ and $y_j = r_0\sin \frac{{j - 1}}{N}{{\Delta }}\alpha$ with ${{\Delta }}\alpha = \frac{\pi }{{90}}$. The orientation angle profile of the metasurface can be calculated by substituting these parameters into Eq. 3.

**Fig. 5:**
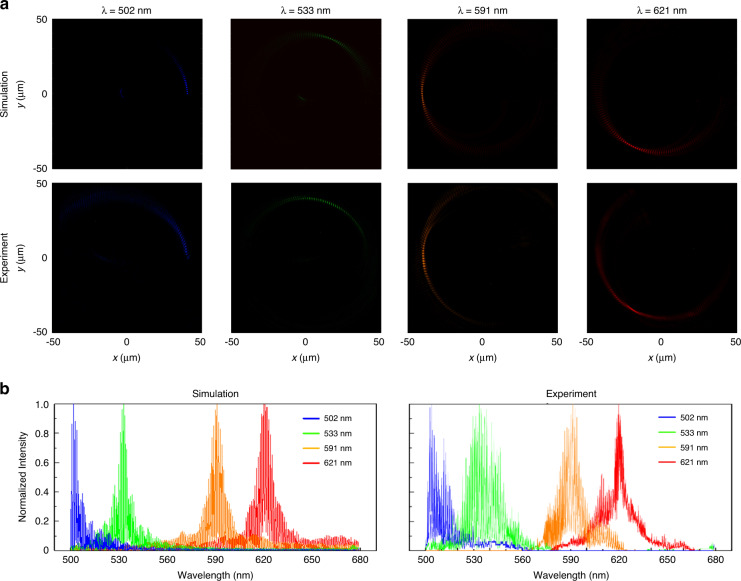
Performance of metasurface spectrometer with 180 focal points under monochromatic wavelengths. **a** Simulation and experimental results at 502 nm, 533 nm, 591 nm, and 621 nm. **b** Normalized intensity distributions on the ring corresponding to the simulation and experimental results in (**a**)

The performance of metasurface with 180 focal points under the monochromatic wavelengths is shown in Fig. 5. Instead of a single focal point, a series of focal points appear in the desired regions on the multi-foci ring. The incident wavelengths are 502 nm, 533 nm, 591 nm, and 621 nm, as shown in Fig. 5a. The presence of multiple focal points instead of a single focal point is due to the small intrinsic dispersion between the focal points. Hence, the intensity of the output light beam is non-zero at these positions. Further details are provided in Supplementary Section 7. An exact incident wavelength can be obtained with high accuracy by analyzing the intensity distribution on the ring. Figure 5b presents the normalized intensity distributions on the ring corresponding to the simulation and experimental results in Fig. 5a. The radius and width of the ring are 40 μm and 0.5 μm, respectively. The maximum value of normalized intensity determines the value of the incident wavelength in the simulation results. Similarly, the incident wavelengths in the experiments can be confirmed by the measured intensity distributions. The measured values of wavelengths at the maximum intensities are 504.1 nm, 533.6 nm, 591.4 nm, and 619.8 nm for the input wavelengths of 502 nm, 533 nm, 591 nm, and 621 nm, respectively. The reasons for the small measurement error can be fabrication errors and a slight misalignment between the sample and the optical path. The values of input wavelength from a supercontinuum laser source are measured with a commercial spectrometer (Ocean Optics FLAME-S) for reference. The detail is provided in Supplementary Section 8. The designed metasurface spectrometer can achieve wavelength detection with a high resolution of 1 nm in a small optical path. Detailed explanation on the resolution of the proposed metasurface spectrometer can be found in Supplementary Section 9. In addition, the simulation and experimental results under the illumination of two boundary wavelengths are provided in Supplementary Section 10. The relative error is less than 0.5% under a stable output of the laser, as predicted by the results. The error in the measurement can be further minimized by achieving near-perfect alignment between the sample and optical path, therefore, can meet the needs of on-chip photonic integration.

**Fig. 6:**
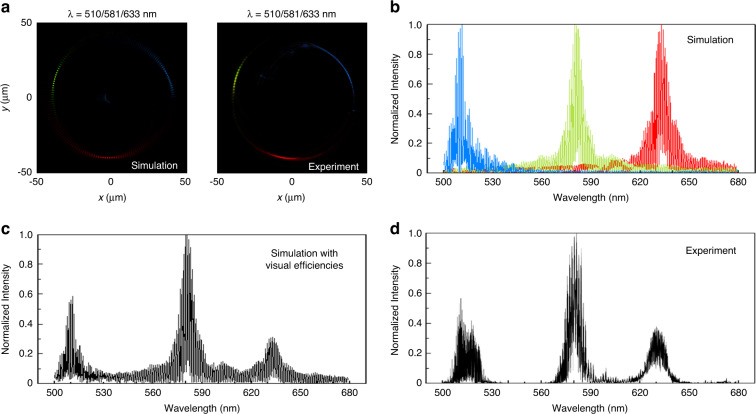
Analysis of metasurface spectrometer with 180 focal points under polychromatic light beams. **a** Simulation and experimental results under a combination of blue, red, and green wavelengths. **b** Normalized intensity distribution on the ring corresponding to the simulation result in (**a**). **c** Simulation results with the consideration of visual efficiencies of the wavelengths. **d** Normalized intensity distribution on the ring corresponding to the experimental result in (**a**). The measured values of peak intensities yield central wavelengths of 511.1 nm, 581.6 nm, and 633.4 nm for the input wavelengths of 510 nm, 581 nm, and 633 nm, respectively

The proposed metasurface spectrometer with 180 focal points is also characterized with a polychromatic incident light beam as shown in Fig. 6. The polychromatic incident light beam consists of 510 nm, 581 nm, and 633 nm wavelengths. The simulation and experimental results of the intensity distribution on the multi-foci ring are presented in Fig. 6a. Three series of focal points appear at the designed positions on the ring. The positions of the focal points with the maximum intensities identify the working wavelengths. For each wavelength, the simulation results of the normalized intensity distribution on the ring are shown in Fig. 6b. For a more accurate prediction, the total intensity distribution on the ring should be compensated for the visual efficiencies of different wavelengths. The visual efficiencies for the wavelengths of 510 nm, 581 nm, and 633 nm are 0.503, 0.860, and 0.235, respectively. The data are obtained from International Commission on Illumination (CIE)^45^. Therefore, the normalized peak intensities after correction are 0.564, 1, and 0.313 at the three wavelengths, as shown in Fig. 6c. The measured result of the normalized intensity distribution on the ring is shown in Fig. 6d. Three spectral profiles with peak intensities of 0.582, 1, and 0.343 are obtained. The measured values of peak intensities yield central wavelengths of 511.1 nm, 581.6 nm, and 633.4 nm for the input wavelengths of 510 nm, 581 nm, and 633 nm, respectively. The relative error of proposed metasurface spectrometer for wavelength identification is still less than 0.5% in the case of a polychromatic incident light beam. The results show that the proposed single-layer design of the metasurface spectrometer has achieved wavelength detection with high-resolution (1 nm) in the case of both mono and polychromatic incident light beams. Unlike conventional spectrometers, a multi-foci metalens functions as a spectrometer. A single metasurface can integrate wavelength splitting and light focusing, which are used to map the wavelength information into the predesigned focal points. We expect that these unique properties of the spectrometer can find exciting applications in the on-chip integrated photonics.

## Discussion

A spectrometer is expected to accurately identify the central wavelength of the light source and detect the linewidth of the spectrum. To fully analyze the functionality and calibrate the proposed metasurface spectrometer, light sources with different linewidths can be used. Laser source with a small linewidth can be an excellent choice in a single frequency operation. Therefore, a common helium-neon laser with linewidth of 10^–6 ^nm is used to obtain the linewidth of the spectrum. When the light beams with a single frequency are determined using the proposed metasurface spectrometer, the difference in respective light intensities is used to obtain the spectral linewidth. Details are provided in Supplementary Section 11. Additionally, the approach can also be used to design a large-area metasurface to detect the linewidth of light beam and even continuous spectrum. Details are provided in Supplementary Section 12 and Supplementary Section 13. At the same time, further increase in the sample size can increase the radius of the multi-foci ring to include more wavelength dependent focal points, which will help enhance the resolution and working bandwidth of the metasurface spectrometer. Detailed explanation is provided in Supplementary Section 14. It is important to note that plasmonic metasurface is used for the proof of concept. The polarization conversion efficiency of metasurface is low which can be improved by employing a dielectric metasurface^46,47^. Alternatively, the reflective-type plasmonic metasurfaces can also be used to achieve a higher conversion efficiency in a broadband domain^48^. Plasmonic metasurface used in this work provides a relatively flat and uniform polarization conversion efficiency in a wide wavelength range as shown in Supplementary Section 2.

In conclusion, we have proposed and experimentally demonstrated a single-layer metasurface spectrometer based on the intrinsic dispersion and multi-foci property of the metalens. The relation between each focal point and the incident wavelength provides a new degree of freedom for spectrometer design. Nanometer spectral resolution over a broadband wavelength in the visible domain has been achieved with the developed ultra-compact metasurface spectrometer. The proposed approach is very flexible and robust, providing a new scheme for controlling the desired dispersion under the illumination of both mono and polychromatic incident light beams. The design flexibility and ultrathin nature render the ultra-compact spectrometer very attractive for monolithic on-chip integration with sensor technology. Therefore, the technology can be effectively utilized for many exciting applications in the areas of spectral analysis, information security, and information processing.

## Methods

Each metasurface sample has an area of 300 × 300 μm^2^ and consists of gold nanorods patterned on top of an ITO coated silicon dioxide (SiO_2_) substrate. The positive polymethyl methacrylate (PMMA) 950 A2 resist is spin coated on the substrate at 1000 rpm for 60 s, producing a PMMA film with a thickness of 120 nm. The sample is baked at approximately 185 °C for 240 s using a hotplate. After that, the electron-beam lithography (Raith PIONEER) is used to pattern the nanostructures on the substrate. During the patterning process, the values of the accelerating voltage and beam current are kept at 30 kV and 12 pA, respectively. Next, the mixture of MIBK:IPA (1:3) is used to develop the exposed patterns by submerging the sample for 50 s. The sample is then immediately immersed in IPA for 40 s. Afterward, the sample is placed in an electron beam evaporator for the deposition of a 40 nm thick gold film. Finally, the metasurfaces are obtained after the lift-off process in the acetone for 10 h.

## Supplementary information

Supplementary Information

Dynamic change of the focal point

## Data Availability

The data that support the findings of this study are available from the corresponding author upon reasonable request.

## References

[1] Staveley, L. A. K. & International Union of Pure and Applied Chemistry, Commission on Physicochemical Measurements and Standards. *The Characterization of Chemical Purity: Organic Compounds* (Butterworths, 1971).

[2] Hilderson, H. *Fluorescence Studies on Biological Membranes*, Vol. 13 (Springer Science & Business Media, 2012).

[3] Zhu AY (2014). Optoelectromechanical multimodal biosensor with graphene active region. Nano Lett..

[4] Schuurs AH, Van Weemen BK (1977). Enzyme-immunoassay. Clin. Chim. Acta.

[5] Wang WB, Paliwal J (2007). Near-infrared spectroscopy and imaging in food quality and safety. Sens. Instrum. Food Qual. Saf..

[6] Zhu AY (2019). Compact aberration-corrected spectrometers in the visible using dispersion-tailored metasurfaces. Adv. Optical Mater..

[7] Grüner-Nielsen L (2000). Dispersion compensating fibers. Optical Fiber Technol..

[8] Chen WT, Zhu AY, Capasso F (2020). Flat optics with dispersion-engineered metasurfaces. Nat. Rev. Mater..

[9] Weiner AM (2000). Femtosecond pulse shaping using spatial light modulators. Rev. Sci. Instrum..

[10] Smith GH, Novak D, Ahmed Z (1997). Overcoming chromatic-dispersion effects in fiber-wireless systems incorporating external modulators. IEEE Trans. Microw. Theory Tech..

[11] Keltner Z (2007). Prism-based infrared spectrographs using modern-day detectors. Appl. Spectrosc..

[12] Cvetojevic N (2012). Developing arrayed waveguide grating spectrographs for multi-object astronomical spectroscopy. Opt. Express.

[13] Faklis D, Morris GM (1995). Spectral properties of multiorder diffractive lenses. Appl. Opt..

[14] Xue QS (2011). Astigmatism-corrected Czerny–Turner imaging spectrometer for broadband spectral simultaneity. Appl. Opt..

[15] Momeni B (2009). Integrated photonic crystal spectrometers for sensing applications. Opt. Commun..

[16] Ferrari M, Quaresima V (2012). A brief review on the history of human functional near-infrared spectroscopy (fNIRS) development and fields of application. Neuroimage.

[17] Bockstaele, R., Luyssaert, B. & Naessens, K. Compact catadioptric spectrometer. China patent CN101548162B (2011).

[18] Grabarnik S (2008). High-resolution microspectrometer with an aberration-correcting planar grating. Appl. Opt..

[19] Wang RX (2021). Metalens for generating a customized vectorial focal curve. Nano Lett..

[20] Chen XZ (2012). Dual-polarity plasmonic metalens for visible light. Nat. Commun..

[21] Khorasaninejad M (2016). Metalenses at visible wavelengths: diffraction-limited focusing and subwavelength resolution imaging. Science.

[22] Wen D (2015). Helicity multiplexed broadband metasurface holograms. Nat. Commun..

[23] Ansari MA (2019). A spin-encoded all-dielectric metahologram for visible light. Laser Photonics Rev..

[24] Ansari MA (2020). Engineering spin and antiferromagnetic resonances to realize an efficient direction-multiplexed visible meta-hologram. Nanoscale Horiz..

[25] Ansari MA (2020). Breaking polarisation-bandwidth trade-off in dielectric metasurface for unpolarised white light. Nanophotonics.

[26] Kim I (2021). Holographic metasurface gas sensors for instantaneous visual alarms. Sci. Adv..

[27] Kim I (2020). Stimuli-responsive dynamic metaholographic displays with designer liquid crystal modulators. Adv. Mater..

[28] Ahmed H (2022). Multichannel superposition of grafted perfect vortex beams. Adv. Mater..

[29] Ming Y (2022). Creating composite vortex beams with a single geometric metasurface. Adv. Mater..

[30] Yue FY (2016). Vector vortex beam generation with a single plasmonic metasurface. ACS Photonics.

[31] Han J (2020). Optical metasurfaces for generation and superposition of optical ring vortex beams. Laser Photonics Rev..

[32] Ahmed H (2022). Optical metasurfaces for generating and manipulating optical vortex beams. Nanophotonics.

[33] Aieta F (2015). Multiwavelength achromatic metasurfaces by dispersive phase compensation. Science.

[34] Chen WT (2018). A broadband achromatic metalens for focusing and imaging in the visible. Nat. Nanotechnol..

[35] Wang SM (2018). A broadband achromatic metalens in the visible. Nat. Nanotechnol..

[36] Faraji-Dana MS (2018). Compact folded metasurface spectrometer. Nat. Commun..

[37] Gao NH (2018). Ultra-dispersive anomalous diffraction from Pancharatnam-Berry metasurfaces. Appl. Phys. Lett..

[38] Khorasaninejad M (2016). Super-dispersive off-axis meta-lenses for compact high resolution spectroscopy. Nano Lett..

[39] Zhu AY (2017). Ultra-compact visible chiral spectrometer with meta-lenses. APL Photonics.

[40] Li K (2017). Dispersion controlling meta-lens at visible frequency. Opt. Express.

[41] McClung A, Mansouree M, Arbabi A (2020). At-will chromatic dispersion by prescribing light trajectories with cascaded metasurfaces. Light Sci. Appl..

[42] Faraji-Dana MS (2019). Hyperspectral imager with folded metasurface optics. ACS Photonics.

[43] Zang XF (2019). A multi-foci metalens with polarization-rotated focal points. Laser Photonics Rev..

[44] Intaravanne Y (2022). Color-selective three-dimensional polarization structures. Light Sci. Appl..

[45] CIE. CIE 1931 colour-matching functions, 2 degree observer. 10.25039/CIE.DS.xvudnb9b (CIE, 2018).

[46] Devlin RC (2016). Broadband high-efficiency dielectric metasurfaces for the visible spectrum. Proc. Natl Acad. Sci. USA.

[47] Chen BH (2017). GaN metalens for pixel-level full-color routing at visible light. Nano Lett..

[48] Zheng GX (2015). Metasurface holograms reaching 80% efficiency. Nat. Nanotechnol..

