# Supplementary material for: Compact multi-foci metalens spectrometer
Source: Light Sci Appl. 2023 May 4;12:103. doi: 10.1038/s41377-023-01148-9 (PMC10160045; doi:10.1038/s41377-023-01148-9)
Supplement: Supplementary file 1 — Supplementary Information [file 41377_2023_1148_MOESM1_ESM.docx]

Supplementary Information

Compact multi-foci metalens spectrometer

**Ruoxing Wang^1, ‡^, Muhammad Afnan Ansari^2, ‡^, Hammad Ahmed^2^, Yan Li^2, 3^, Wenfeng Cai^4^, Yanjun Liu^4^, Songtao Li^1^, Jianlong Liu^5^, Li Li^6^, and Xianzhong Chen^2,^ ***

*^1^Department of Mathematics and Physics, North China Electric Power University, Baoding, 071003, China*

*^2^Institute of Photonics and Quantum Sciences, School of Engineering and Physical Sciences, Heriot-Watt University, Edinburgh, EH14 4AS, UK*

*^3^School of Materials, Zhengzhou University of Aeronautics, Zhengzhou, 450015, China*

*^4^Department of Electrical and Electronic Engineering, Southern University of Science and Technology, Shenzhen, 518055, China*

*^5^College of Physics and Optoelectronic Engineering, Harbin Engineering University, Harbin, 150001, China*

*^6^School of Physics, Harbin Institute of Technology, Harbin, 150001, China*

* Corresponding Authors

Email: x.chen@hw.ac.uk

**^‡^** These authors contributed equally: Ruoxing Wang, Muhammad Afnan Ansari

**Keywords:** metasurface spectrometer, muti-foci metalens, wavelength detection, dispersion control.

**Supplementary Section 1. Control of off-axis focal spots using new multiwavelength and** **multi-foci metalens design**

Single off-axis focal points with different incident wavelengths are illustrated in **Figure S1**. Every focal point generated by the multi-foci metalens is placed at a focal plane *f* = 300 μm and 30 μm off-axis with respect to the optical axis. It is noted that all focal points are approximately equal in size and possess almost the same focal depth with maximum intensity on the designed focal plane. The focusing condition of each focal point is the same at different wavelengths on the ring. This is due to the inclusion of additional wavelength information in the phase profile in **Equation 3** and an equal distance from the center of the metasurface to the focal points. Therefore, the ring-based design is preferable compared to a flat design. This functionality has not been realized in the previously demonstrated metalens designs with off-axis focal points ^1,2^.

**Figure S1.** Focal spots of the designed multi-wavelength and multi-foci metalens under the illumination of light beams with different wavelengths. It demonstrates the nearly equal size and focal depth at a single focal plane. Under all incident light beams, the beam waist and the focal plane remain the same due to the inclusion of an extra design variable in the multi-foci metalens design, i.e., wavelength.

The theoretical variation of focal point size as a function of wavelength is shown in **Figure S2**. The focal point diameter *d*_0_ of lens satisfies the classical formula *d*_0_ = 2*λ*_0_*f* / *D*, where *D* is the clear aperture. The focal spot contains 86.5% of the focusing energy. For our sample design, the actual focal distance of each focal point is *f_D_*, and the value of the clear aperture is equal to the diameter of the sample. In simulation, the radius of focal spot is slightly smaller than the theoretical value. This is because the center intensity is chosen to drop to e^-2^ (13.5%) as the focal spot radius instead of the focusing energy containing 86.5%. As the size of the focal spot is approximately equal to 1 μm and the detection area is equal to 100×100 μm^2^, therefore, all focal points look approximately equal in size on the detection area.


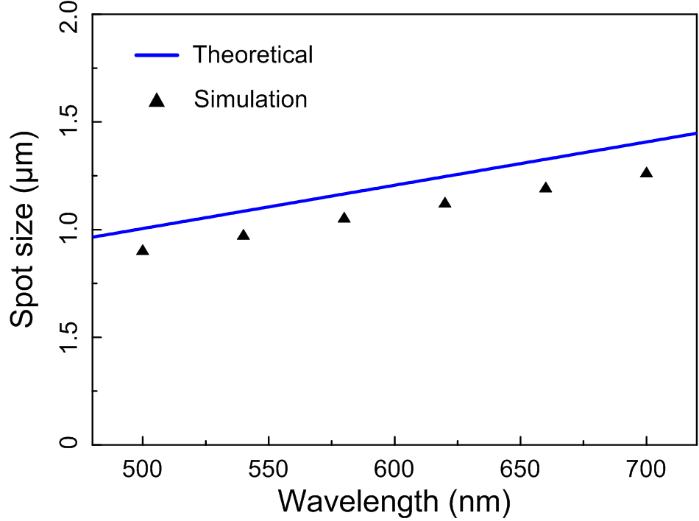


**Figure S2**. Focal point size as a function of wavelength.

**Supplementary Section 2. Conversion efficiency of the designed** **metasurface spectrometer**

The designed metasurface spectrometers are realized based on plasmonic metasurfaces, which consist of gold (Au) nanorods with spatially variant orientations sitting on a glass substrate (SiO_2_) as shown in **Figure S3a**. Each nanorod is *l* = 200 nm long, *w* = 80 nm wide, and *t* = 40 nm high. The periodicity (*p*) of each unit cell is 300 nm along both *x* and *y* directions. The simulated conversion efficiency is shown in **Figure S3b**. The efficiency is calculated using the frequency domain solver of the Computer Simulation Technology (CST) Microwave Studio software. In the simulation, the permittivity of gold nanorod is calculated by the Drude model with the plasma frequency *ω_p_* = 1.37 × 10^16^ rad s^-1^, and the collision frequency *γ_c_* = 1.215 × 10^14^ rad s^-1^. The refractive index of the glass substrate is 1.46. The unit cell boundary is used along the x and y directions, and the open boundary condition is used along the z direction. The calculated conversion efficiency is relatively flat and uniform within the design band ranging from 480 nm to 700 nm, with the conversion efficiency exceeding 8%. Although the converted part is low and the non-converted part is extremely high, it can be filtered out by using the experimental setup in **Figure 2c**. The reflective plasmonic metasurfaces can achieve a higher conversion efficiency in broadband. Dielectric metasurfaces can dramatically improve efficiency ^3-5^, which can be optimized to achieve a broadband design for metasurface spectrometers.


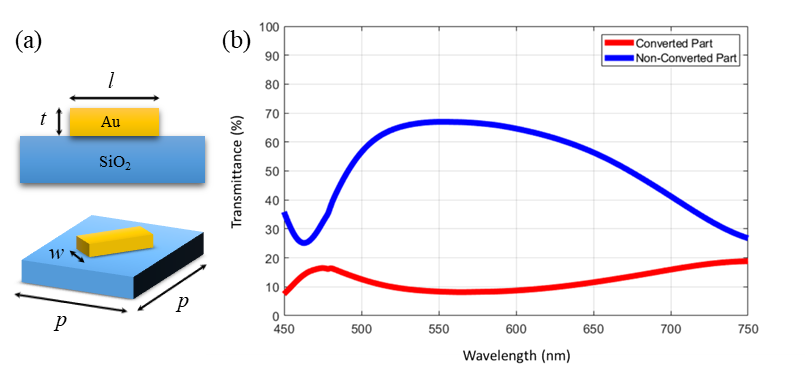


**Figure S3.** (a) Metaatom design. (b) Simulated non-converted and converted parts of the transmittance spectra in the visible region.

**Supplementary Section 3. Effect of cone angle of the incident beam on focusing performance of the metalens**

The proposed metasurface was designed at normal incidence. To increase the power density of our supercontinuum laser source, we used a lens to weakly focus the light beam on the metasurface. The incident light beam has a cone angle which slightly changes the focal length of multi-foci metalens. The larger cone angle will focus the light beam closer to the metasurface. We have used a lens with focal length *f* = 100 mm. The cone angle of the lens is equal to 0.57°. If we use the lens with a smaller *f* = 75 mm, the cone angle will become slightly larger (0.76°) and focal length of multi-foci metalens will slightly decrease. If we use the lens with a larger *f* = 150 mm, the cone angle will become smaller (0.38°) and focal length of multi-foci metalens will slightly increase. However, the effect of small cone angle on the field distribution on the focal plane of metalens is negligible due to the paraxial approximation as shown in **Figure S4**. The experimental results are obtained with a sample having 12 focal points under 660 nm wavelength.


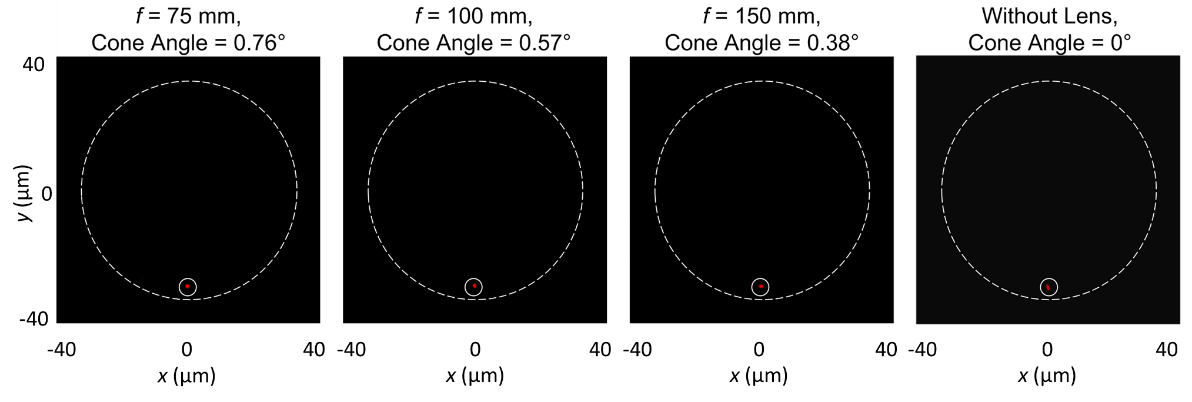


**Figure S4.** Effect of cone angle generated by the L1 on the focusing performance of the metalens. The involvement of L1 can introduce a cone angle of the incident beam. The incident beam with a smaller cone angle can produce results that are much closer to the ideal normal incidence.

**Supplementary Section 4. Description of Additional Supplementary Files**

**File name: Movie 1**

Description: Metasurface spectrometer that can realize the dynamic change of designed focal points as the incident wavelength is changed from 500 nm to 700 nm.

**Supplementary Section 5. Focal points of metasurface spectrometer under the incident wavelengths away from the designed wavelengths**

The behavior (depicted in **Figure S5**) exists due to a relatively small intrinsic dispersion. For the focal point of a smaller designed wavelength, the light beam is converged after the focal plane. In contrast, for a focal point of a higher-designed wavelength, the light beam is converged in front of the focal plane. More details can be found in **Supplementary Section 7**. The experimental results in **Figures S5d** and **S5e** are slightly different from the simulation results because the calculated intensity distribution is the result on the focal plane. However, the experimentally observed pattern is obtained within a small depth of field.


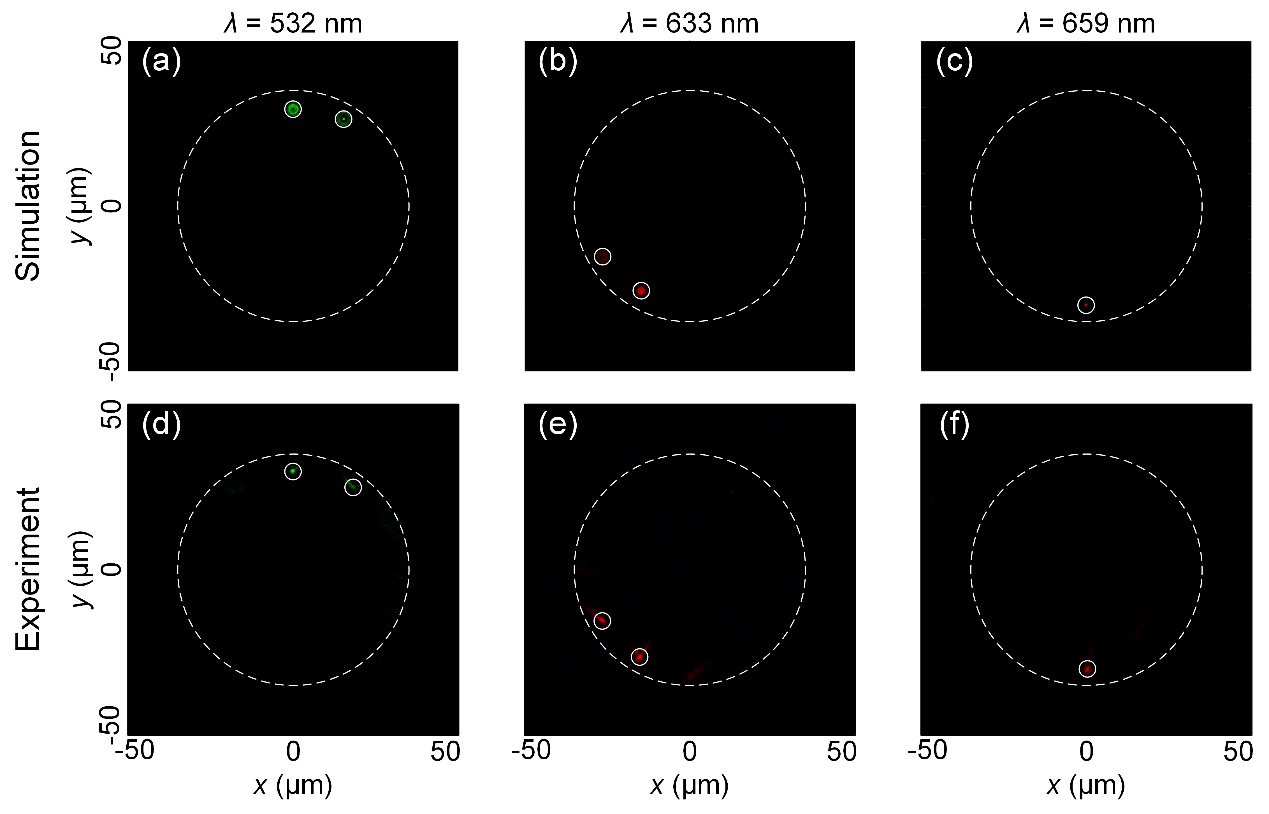


**Figure S5.** Metasurface spectrometer with 12 focal points under monochromatic wavelengths other than working wavelengths. Simulation results at (a) 532 nm, (b) 633 nm, and (c) 659 nm. Experimental results at (d) 533.3 nm, (e) 633 nm, and (f) 659 nm.

**Supplementary Section 6.** **Effect of multi-foci** **ring radius on the dispersion difference between focal points**

When the working wavelengths are designed to be 1 nm apart with a range from 500 nm to 679 nm (*N* = 180), the radius of the multi-foci ring plays a critical role in the distribution of focal points on the focal plane. **Figure S6** presents the simulation results of the field distributions with different radii at 545 nm incident wavelength. If the radius is too small, the number of 180 focal points will lead to an overlap of the adjacent focal positions. As shown in **Figure S6a**, the designed focal positions will be difficult to distinguish due to the overlap and strong diffraction. When the radius is increased, the focal positions are easily recognized, as shown in **Figures S6b** and **S6c**. It is also noted that the distance between the adjacent focal positions becomes wider with a higher dispersion difference. More results with different values of the radius are provided in **Supplementary Section 12**. To improve the wavelength detection accuracy, $r_{0}$ = 40 μm is chosen to design and fabricate the second sample. It ensures that within the same observation area as the first sample in the main text, the adjacent focal positions can obtain a higher dispersion difference and be easily observed.

**Figure S6.** Simulation results for the same number of focal spots with varying ring radius for higher dispersion difference at 545 nm incident wavelength. The radius $r_{0}$ is equal to (a) 30 μm, (b) 35 μm, and (c) 40 μm. A small radius means more overlap and low dispersion difference between focal points of adjacent wavelengths compared to the design with a higher radius. To improve the wavelength detection capability within the same observation area, a higher dispersion difference between focal points of adjacent wavelengths is required. Therefore, the radius of the ring equal to 40 μm is selected for the design.

**Supplementary Section 7. Effect of adjacent incident wavelengths on the focusing condition of focal point**

The combined phase profile of the metasurface has the information of all working wavelengths. For instance, in case of a monochromatic incident light beam, only one focal point will converge exactly on the focal plane at the corresponding position of its own wavelength, and other focal points will also converge in front or after the designed focal plane at this position. To clarify this, a focal position of 545 nm is chosen with multi-foci ring radius $r_{0}$ = 30 μm as an example. The convergences under different incident wavelengths at this position are shown in **Figure S7**. When the incident wavelength is less than 545 nm, the focal point will converge after the designed focal plane. In contrast, when the incident wavelength is larger than 545 nm, the focal point will converge in front of the focal plane. Due to the small focal depth, the intensity on the focal plane is greatly affected by the adjacent wavelength. The intensity of the focal point become significantly low if the incident wavelength is more than 5 nm away from the designed wavelength position. Thus, instead of a single focal point, a series of focal points appear on the multi-foci ring as discussed in the main text.


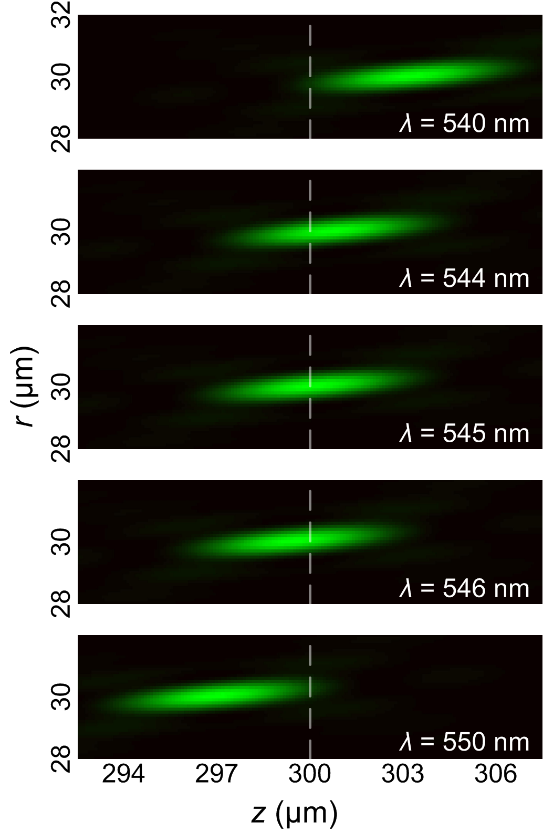


**Figure S7**. Focusing condition of the adjacent incident wavelengths at the designed wavelength of 545 nm. The incident wavelengths from top to bottom are 540 nm, 544 nm, 545 nm, 546 nm, and 550 nm, respectively.

**Supplementary Section 8.** **Wavelength measurement of supercontinuum laser** **by a** **commercial high-precision spectrometer**

**Figure S8** presents the spectrum of a supercontinuum laser measured by a commercial high-precision spectrometer. The supercontinuum laser is set to generate four wavelengths simultaneously. The center wavelengths measured by the commercial high-precision spectrometer are 502 nm, 533 nm, 591nm, and 621 nm.


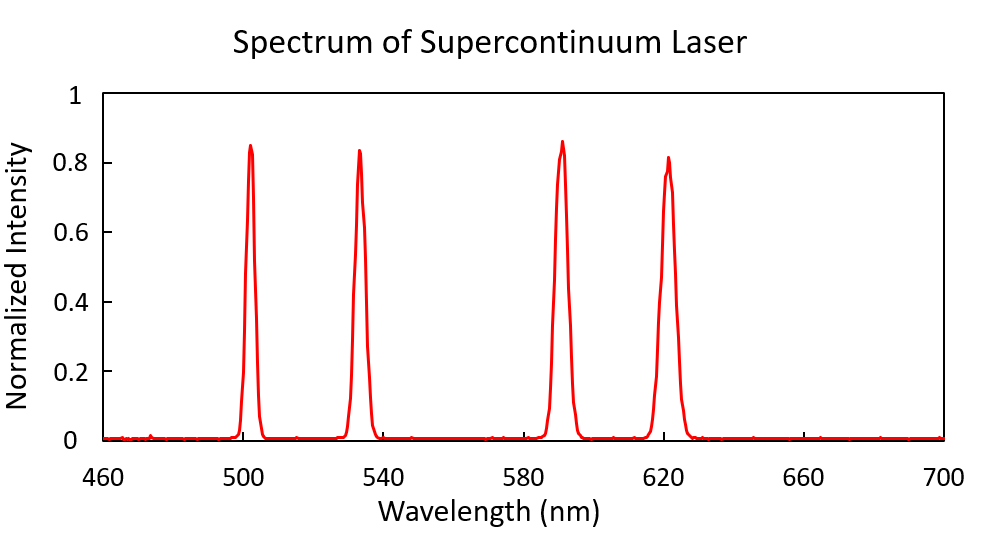


**Figure S8.** The spectrum of the supercontinuum laser measured by a commercial high-precision spectrometer.

**Supplementary Section 9. Ability to distinguish the wavelengths with 1 nm difference**

**Figure S9** shows the simulation and experimental results of the metasurface with 180 focal points at the incident wavelengths of 544 nm, 545 nm, and 546 nm. The three maximum intensities on the multi-foci ring appear at the corresponding incident wavelength focal positions as shown in **Figures S9a** and **S9b**. **Figure S9c** presents the corresponding experiment results, which are in good agreement with the simulation results. Under the illumination of 545 nm, the maximum intensity focal point appears on the vertical centerline on the observation area. Upon the illumination of incident light at 544 nm and 546 nm, the maximum intensity points are located on the right and left side of the vertical centerline, respectively. This proves that the proposed metasurface spectrometer can accurately identify and distinguish the wavelengths with 1 nm difference.


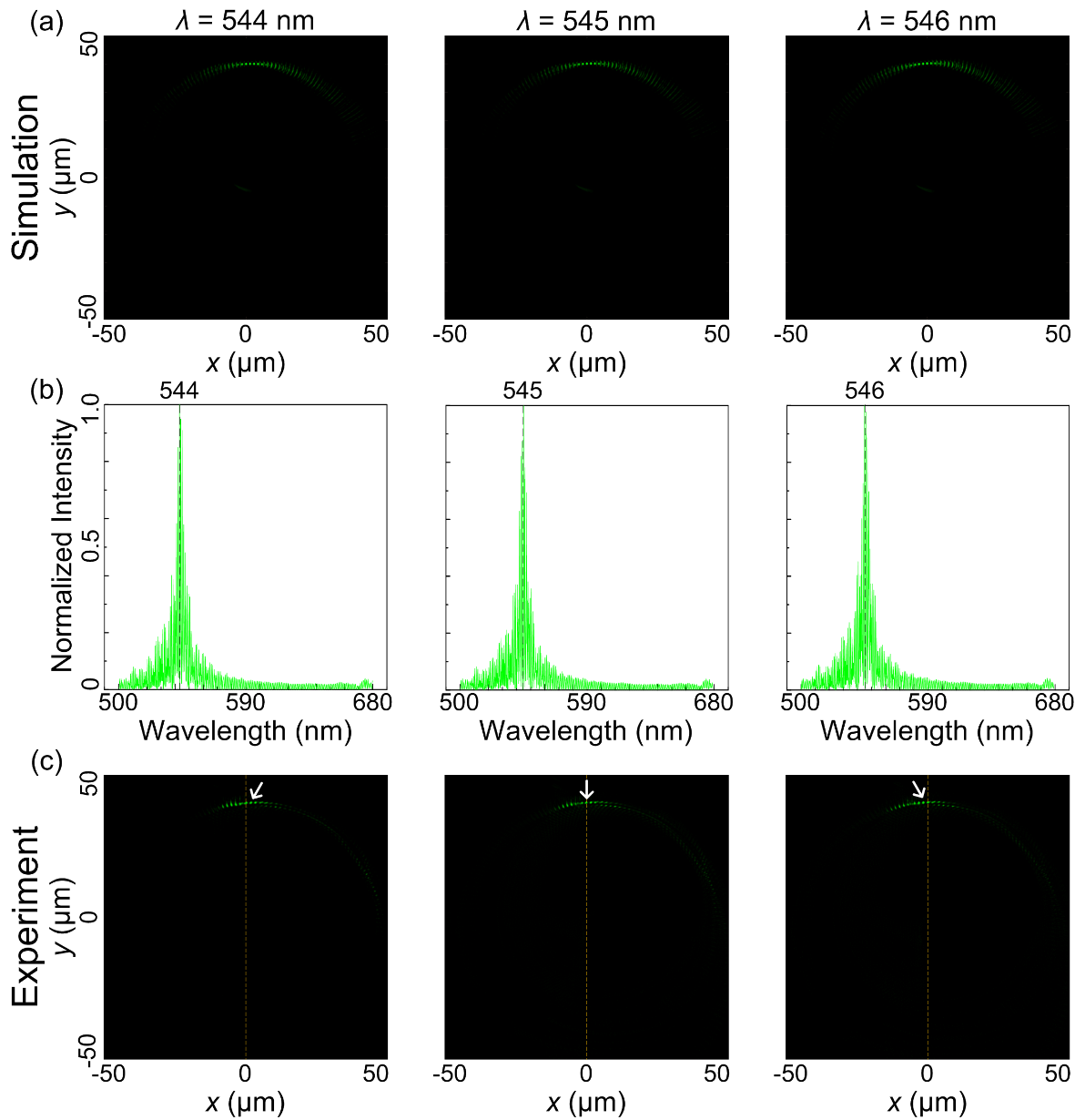


**Figure S9**. (a) Simulation of the field distribution under the illumination of incident light at 544 nm, 545 nm, and 546 nm. (b) Normalized intensity distributions on the multi-foci ring. (c) Experimental results.

**Supplementary Section 10. Metasurface spectrometer under the illumination of two boundary wavelengths**

**Figure S10** presents the simulated and measured performance of metasurface with 180 focal points under the illumination of two boundary wavelengths, i.e., 500 nm and 679 nm. It is noted that the two boundary wavelengths are accurately detected by the metasurface spectrometer. The high intensity focal points are located at the desired positions, i.e., 0° and 358° for 500 nm and 679 nm, respectively. Even though their focal positions are very close, due to the large wavelength difference, the large intrinsic dispersion is not enough to converge one boundary wavelength at the other boundary wavelength focal position, which ensures the effectiveness of the spectrometer in the whole designed wavelength band. As shown in **Figure S10b**, the experiment results are consistent with the simulation results. Under the incidence of 500 nm, the focal point with maximum intensity appears on the horizontal centerline, and the remaining focal points are located above the horizontal centerline. Under the incidence of 679 nm, all focal points are located below the horizontal centerline and the maximum intensity point is at the desired position which is close to the horizontal centerline.


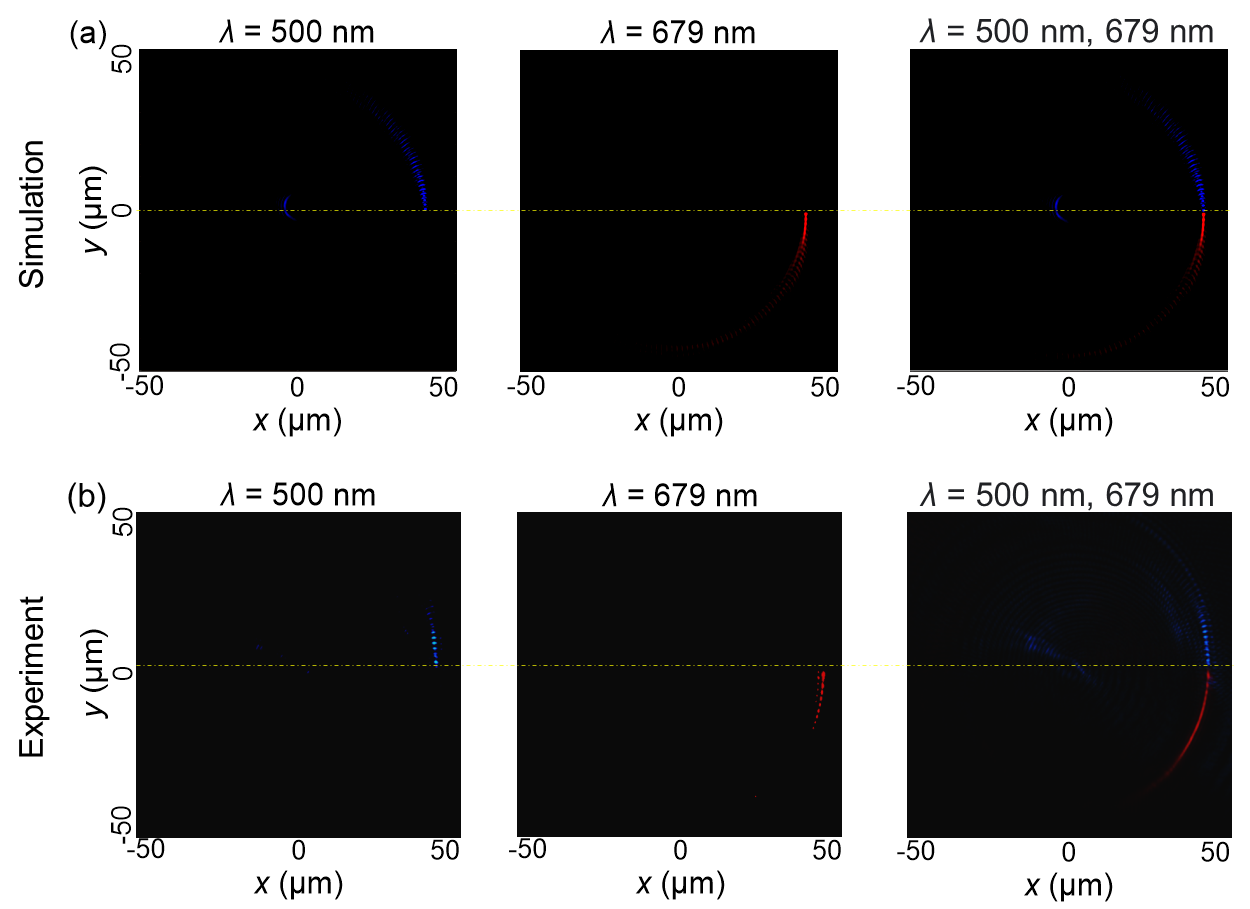


**Figure S10**. Performance of the metasurface spectrometer at two boundary wavelengths. (a) Simulation and (b) experimental results at a single wavelength (500 nm or 679 nm) and two wavelengths (500 nm and 679 nm).

**Supplementary Section 11. Principle of measuring spectral** **linewidth with our designed metasurface spectrometer**

A spectrometer is expected to not only accurately identify the central wavelength of the light source, but also to detect the linewidth of the spectrum. In order to obtain the linewidth of the spectrum, we propose an ideal single-frequency light beam and light sources with different linewidths to calibrate the metasurface spectrometer. Here, a new sample is designed with working wavelengths having a 1 nm interval and range from 621 nm to 640 nm (*N* = 20). The designed working wavelength of the *j*th focal point is $\lambda_{j}=\lambda_{0}+(j-1)\Delta\lambda$, where $\lambda_{0}$ = 621 nm, $\Delta\lambda$ = 1 nm, and *j* = 1 to 20 (integer number). All focal points are located on a ring with a radius of $r_{0}$ = 40 μm on the focal plane *f* = 300 μm, and the coordinates of each focal point are $x_{j}=r_{0}\cos\frac{j-1}{N}\Delta\alpha$ and $y_{j}=r_{0}\sin\frac{j-1}{N}\Delta\alpha$, where $\Delta\alpha=\frac{\pi}{10}$. With these parameters, the phase profile of the new sample can be obtained based on **Equation 3** in the main text. **Figures** **S11a-c** present the simulation results of field distributions on the focal plane at 633 nm with different linewidths, where the FWHM are (**a**) 1 nm, (**b**) 2 nm, and (**c**) 0 nm (ideal laser light source), respectively. It is difficult for the naked eye to distinguish the difference between the field distributions. However, there exists a difference in the intensity distribution on the multi-foci ring between a light source with a linewidth and an ideal laser light source. The intensity differences in the multi-foci ring between the light sources with linewidths (FWHM = 1 nm and 2 nm) and the ideal laser source are shown in **Figures S11d** and **S11e**. The maximum values of the intensity differences between light sources with different linewidths and ideal laser source are different. In theory, a higher linewidth value will bring a higher maximum difference value. Therefore, by pre-calibrating the difference generated by different sources, the linewidth can be analyzed by measuring the intensity difference on the multi-foci ring with an ideal laser source. In the experiment, a helium-neon (He-Ne) laser is used as a light source with a linewidth of less than 10^-6^ nm. The experimental result of the field distribution on the focal plane is shown in **Figure S11f**. The experimentally measured field distribution on the focal plane under the light beam of a supercontinuum laser with a 633 nm wavelength is shown in **Figure S11g**. However, the linewidth of the supercontinuum laser at 633 nm was not obtained due to the resolution and sensitivity limitations of our CCD camera. The linewidth can be measured using the aforementioned method with a high-resolution CCD camera sensitive to 1% change in the intensity of the light beam.

**Figure S11**. (a)-(c) Simulation results of the normalized field distribution on the focal plane under the incident light beam of 633 nm with different linewidths. The FWHM is (a) 1 nm, (b) 2 nm, and (c) 0 nm for the ideal laser source. Intensity differences between the light source with linewidths and the ideal laser source for FWHM (d) 1 nm, and (e) 2 nm. Experimental results of the field distributions with (f) He-Ne laser source and (g) supercontinuum laser source at 633 nm.

**Supplementary Section 12. Measuring the spectral linewidth with a larg****e metasurface**

Initially in **Supplementary Section 6**, the effect of multi-foci ring radius on the dispersion difference between focal points is studied in a small range to ensure that the experiment can be carried out in the same observation area. Here, the effect of change due to multi-foci ring radius in a higher range is studied. The working wavelengths of the metasurface spectrometer are 1 nm apart from 500 nm to 679 nm (*N* = 180) with higher radius values. The simulation results of the field distribution with different radius values (40 μm, 80 μm, 120 μm, 160 μm and 200 μm) at 545 nm incident wavelength are shown in **Figure S12a**. The normalized intensity distribution on each ring is shown in **Figure S12b**. The width of the ring is taken as 0.5 μm. As the radius increases, the distance between the designed adjacent focal positions becomes higher with a higher dispersion difference.

**Figure S12.** (a) Simulation results of the field distribution with different radius values (40 μm, 80 μm, 120 μm, 160 μm, and 200 μm) at 545 nm incident wavelength, and (b) corresponding normalized intensity distributions on each multi-foci ring.

The case of a further increase in the value of the radius of multi-foci ring (300 μm, 400 μm, 500 μm, and 600 μm) at 545 nm incident wavelength are shown in **Figure S13a**. The corresponding normalized intensity distributions on each ring are shown in **Figure S13b**. In this case, the further increase in the radius of the ring causes a decrease in the dispersion difference between the designed adjacent focal positions. This is because the multi-foci radius is increased to an off-axis distance which is more than the radius of the metalens aperture. Therefore, the focal distance increases significantly and results in an increase in the focal size. It also affects the dispersion difference at the designed adjacent focal positions.

**Figure S13.** Decrease in dispersion difference due to large values of ring radius. (a) Simulation results of the field distributions with different radius values (300 μm, 400 μm, 500 μm, and 600 μm) at 545 nm incident wavelength, and (b) corresponding normalized intensity distributions on each multi-foci ring.

The focal spot size is determined by the focal length and clear aperture. Therefore, the size of the focal spot with a large focal distance can be reduced by increasing the clear aperture. Here, the effect of a clear aperture on the dispersion difference between focal points is also studied. The working wavelengths of the metasurface spectrometer are 1 nm apart, ranging from 500 nm to 679 nm (*N* = 180) with multi-foci radius $r_{0}$ = 600 μm. The total number of pixels of the metasurface spectrometer is defined as *Np*×*Np*. With different values of *Np* (1000, 1200, 1500, and 2000), the field distributions on the focal plane at 545 nm incident wavelength are shown in **Figure S14a**. With the increase of *Np*, the size of the focal spot becomes smaller. The corresponding normalized intensity distributions on each ring are shown in **Figure S14b**. With the increase in *Np*, the dispersion difference at the designed adjacent focal positions becomes larger. When *Np* exceeds 1500, the normalized intensity at the adjacent focal positions is less than 0.05. When *Np* = 2000, dispersion has a less effect on the intensity at the designed adjacent focal positions.

**Figure S14.** (a) Simulation results of the field distributions with different values of total pixels *Np*×*Np* (*Np* = 1000, 1200, 1500, and 2000) at 545 nm incident wavelength, and (b) corresponding normalized intensity distributions on each multi-foci ring.

The effect of dispersion on the intensity at the adjacent focal positions is negligible with multi-foci radius $r_{0}$ = 600 μm and *Np* = 2000 for the working wavelengths. In this case, the simulation results of field distributions at a wavelength of 545 nm with different linewidths (FWHM = 1 nm, 2 nm, and 3 nm) are shown in **Figure S15a**. The normalized intensity distributions on each ring are shown in **Figure S15b** (black line). The incident Gaussian spectrums with different linewidths are also shown in **Figure S15b** (blue line). Moreover, Gaussian fitting is carried out according to the intensity of each designed focal position, and the spectral results are shown in **Figure S15b** (red line). The calculated FWHMs are 1.25 nm, 2.12 nm, and 3.05 nm, respectively, which are very close to their respective incident linewidths. Therefore, based on our approach, a metasurface spectrometer with a higher number of pixels and a higher multi-foci radius will be more helpful in the detection of linewidth of the light beam.

**Figure S15.** Measurement of the linewidth using a large metasurface. (a) Simulation results of the field distributions at a wavelength of 545 nm with different linewidths (FWHM = 1 nm, 2 nm, and 3 nm). (b) Corresponding normalized intensity distributions on each multi-foci ring (black line), incident Gaussian spectrums with different linewidths (blue line), and Gaussian fitting spectral results according to the intensity of each designed focal position (red line).

**Supplementary Section 13. Measuring continuous spectrum with a large-area metasurface**

The feasibility of spectral linewidth detection is demonstrated in **Supplementary Section 12** as shown in **Figure S15**. In the same way, it is possible to measure a real object with a continuous spectrum using proposed multi-foci metalens design. The simulation results of the metasurface spectrometer for three kinds of continuous spectral detection from 543 nm to 547 nm are shown in **Figure S16**, with multi-foci radius $r_{0}$ = 600 μm and *Np* = 2000. The simulation results of the field distributions under three different incident spectral profiles are shown in **Figure S16a**. The three kinds of continuous spectral profiles are shown in **Figure S16b** (blue line). The normalized intensity distributions on each ring are shown in **Figure S16b** (black line). According to the intensity of each designed focal position, the spectral results can be obtained by fitting these intensity values as shown in **Figure S16b** (red line). Therefore, it is evident that a metasurface spectrometer with a higher resolution, higher number of pixels and larger multi-foci radius will promote the accurate detection of continuous spectrum. The current designed resolution of metasurface spectrometer presented in the main manuscript cannot cover the discrete accuracy of continuous spectrum due to its small size. However, as predicted by the simulation results, the detection of real object with continuous spectrum using large metasurface spectrometer is possible.


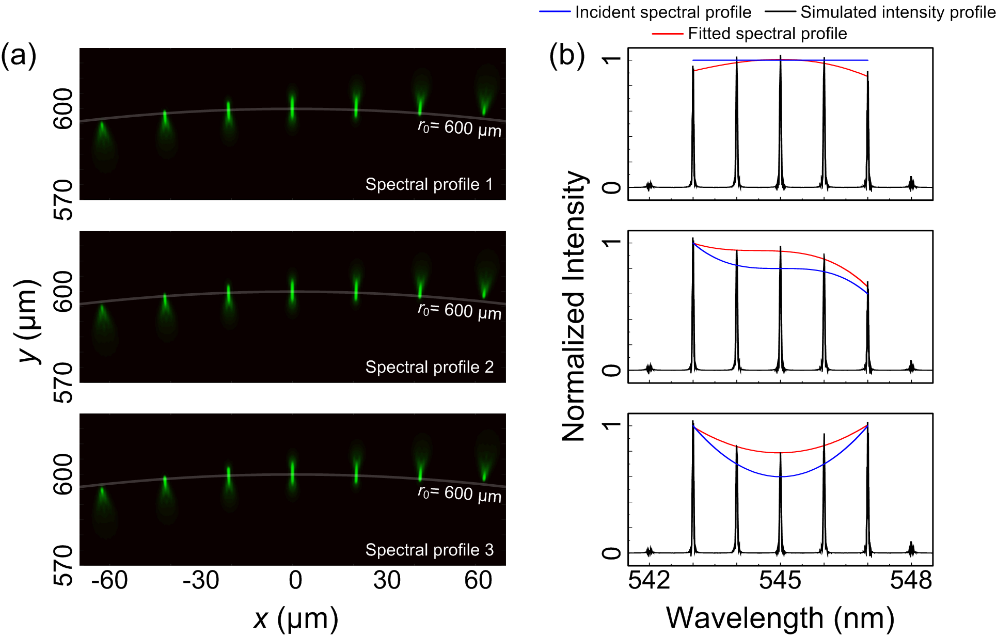


**Figure S16**. Simulation results of the continuous spectrum measurement using a large metasurface. (a) Simulation results of the field distributions under three different incident spectral profiles. (b) Corresponding incident spectral profile (blue line), normalized intensity distributions on each multi-foci ring (black line) and fitted results according to the intensity of each designed focal position (red line).

**Supplementary Section 14. Maximum number of wavelengths of the metasurface spectrometer**

In theory, the maximum number of wavelengths of the metasurface spectrometer that can be distinguished is equal to the circumference of the multi-foci ring divided by the size of the focal spot. For example, if the working wavelength of all focal points on the ring is 590 nm and the multi-foci ring radius is 40 μm, the maximum number of focal points that can be distinguished is 242, as shown in **Figure S17**. In this case, the focal points on the ring are connected with each other without an overlap. However, the overlapping will occur in case of more than 242 focal points on the ring. If the number of focal points is reduced, the spacing between the focal points will become larger, and each focal point will be easier to distinguish. Now, if a different working wavelength is considered for each focal point, the multi-foci ring radius, dispersion difference and metasurface size put a limit on the value of Δ*λ*. Decreasing Δ*λ* means higher resolution, however, for the same ring radius, the dispersion difference between adjacent focal points will decrease. A decrease in Δ*λ* will require a large area metasurface for a bigger ring radius and to compensate for higher dispersion difference between adjacent focal points. According to this tradeoff and our experimental processing conditions, the maximum wavelength number that can be distinguished with current design (Δ*λ* = 1 nm) is 180 ranging from 500 nm to 679 nm. Note that, the further increase in the size of metasurface can achieve larger multi-foci ring radius and accommodate more wavelength dependent focal points. These wavelength dependent focal points can have a larger wavelength range and a smaller Δ*λ*, which will help enhance the resolution and working bandwidth of the metasurface spectrometer.


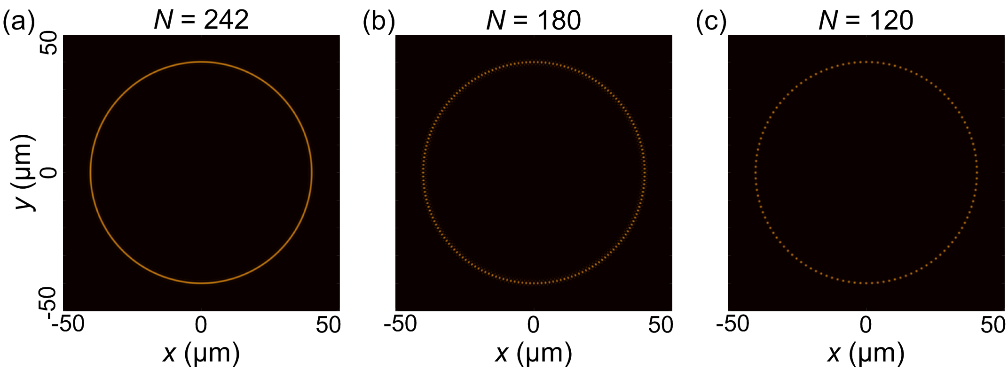


**Figure S17**. Field distributions with different numbers of focal points. The working wavelength of each position is 590 nm, and the radius of the multi-foci ring is 40 μm. The number of focal points on the ring is (a) 242, (b) 180 and (c) 120, respectively.

**References**

1. Zhu, A. Y. et al. Ultra-compact visible chiral spectrometer with meta-lenses. *APL Photonics* **2**, 036103 (2017).

2. Khorasaninejad, M., Chen, W. T., Oh, J. & Capasso, F. Super-Dispersive Off-Axis Meta-Lenses for Compact High Resolution Spectroscopy. *Nano Letters* **16**, 3732–3737 (2016).

3. Chen, B. H. et al. GaN Metalens for Pixel-Level Full-Color Routing at Visible Light. *Nano Letters* **17**, 6345–6352 (2017).

4. Devlin, R. C., Khorasaninejad, M., Chen, W. T., Oh, J. & Capasso, F. Broadband high-efficiency dielectric metasurfaces for the visible spectrum. *Proceedings of the National Academy of Sciences* **113**, 10473–10478 (2016).

5. Ansari, M. A. et al. A Spin-Encoded All-Dielectric Metahologram for Visible Light. *Laser & Photonics Reviews* **13**, 1900065 (2019).
